# Study on gene expression in the liver at various developmental stages of human embryos

**DOI:** 10.3389/fcell.2024.1515524

**Published:** 2025-01-08

**Authors:** Hanqing Chen, Tingting Li, Ming Cai, Zhiqi Huang, Jianjun Gao, Hongping Ding, Minmin Li, Weiyu Guan, Jinpeng Chen, Wenran Wang, Chunhong Li, Jianwu Shi

**Affiliations:** ^1^ Basic Medical Research Centre, Medical School, Nantong University, Nantong, Jiangsu, China; ^2^ Department of Critical Care Medicine, Nantong Third People’s Hospital, Nantong, Jiangsu, China; ^3^ Department of Thyroid and Breast Surgery, Nantong First People’s Hospital, Affiliated Hospital 2 of Nantong University, Nantong, Jiangsu, China; ^4^ Department of Critical Care Medicine, Nantong Second People’s Hospital, Nantong, Jiangsu, China; ^5^ Department of Endocrinology, Third People’s Hospital of Rugao, Nantong, Jiangsu, China; ^6^ Department of Pediatrics, Affiliated Hospital of Nantong University, Nantong, Jiangsu, China; ^7^ Department of General Surgery, Nantong First People’s Hospital, Affiliated Hospital 2 of Nantong University, Nantong, Jiangsu, China

**Keywords:** livers, human embryos, transcriptomic sequencing, development, gene expression

## Abstract

**Background:**

The normal development of the liver during human embryonic stages is critical for the functionality of the adult liver. Despite this, the essential genes, biological processes, and signal pathways that drive liver development in human embryos remain poorly understood.

**Methods:**

In this study, liver samples were collected from human embryos at progressive developmental stages, ranging from 2-month-old to 7-month-old. Highly expressed genes and their associated enrichment processes at various developmental stages of the liver were identified through transcriptomic sequencing.

**Results:**

The findings indicated that genes associated with humoral immune responses and B-cell-mediated immunity were highly expressed during the early developmental stages. Concurrently, numerous genes related to vitamin response, brown adipocyte differentiation, T cell differentiation, hormone secretion, hemostasis, peptide hormone response, steroid metabolism, and hematopoietic regulation exhibited increased expression aligned with liver development. Our results suggest that the liver may possess multiple functions during embryonic stages, beyond serving hematopoietic roles. Moreover, this study elucidated the complex regulatory interactions among genes involved in lymphocyte differentiation, the regulation of hemopoiesis, and liver development. Consequently, the development of human embryonic liver necessitates the synergistic regulation of numerous genes. Notably, alongside conventionally recognized genes, numerous previously uncharacterized genes involved in liver development and function were also identified.

**Conclusion:**

These findings establish a critical foundation for future research on liver development and diseases arising from fetal liver abnormalities.

## Introduction

The liver consists of more than 20 distinct cell types, such as hepatocytes, biliary ductal cells (cholangiocytes), and liver endothelial cells, among others (Robinson et al., 2016; MacParland et al., 2018; Aizarani et al., 2019; Yang et al., 2019). Liver dysfunction, caused by conditions such as fatty liver and liver fibrosis, can trigger a range of pathological symptoms within the human body. It has been reported that the liver plays critical and distinct roles in the fetus and adult. During mid to late gestation, the fetal liver serves as the primary site for hematopoiesis and lacks most metabolic functions, in contrast to the adult liver (Timens and Kamps, 1997). However, this claim warrants further investigation.

During embryonic development, the liver originates from the lateral domain of the endoderm within the ventral foregut (Chalmers and Slack, 2000; Tremblay and Zaret, 2005). Following the specification of foregut endoderm, hepatoblasts bud into the septum transversum, where they continue to proliferate and differentiate. During embryogenesis, hepatocytes and cholangiocytes, the two primary lineages responsible for most liver functions, arise from bipotential hepatoblasts. These progenitor cells are generally believed to originate from the definitive endoderm approximately 3–4 weeks post-coitum in humans and at embryonic day 8.5–9.0 in mice (Gordillo et al., 2015). Maturation into hepatocytes and bile duct epithelial cells continues for several weeks postnatally.

Numerous genes and signaling pathways involved in liver development have been identified. During hepatic specification, WNT signaling facilitates hepatic induction, promoting the emergence and differentiation of liver bud (Finley et al., 2003; Monga et al., 2003; Ober et al., 2006; McLin et al., 2007). At this stage, the newly specified hepatic cells, known as hepatoblasts, adopt a columnar morphology and invade the septum transversum mesenchyme, initiating liver bud formation (Bort et al., 2006). Several transcription factors, including FOXA1/2, GATA4/6, HHEX, and HNF1A/1B, play integral roles in hepatoblast specification (Gordillo et al., 2015; Villasenor and Stainier, 2017). As hepatoblasts begin budding into the surrounding mesenchyme, they continue to proliferate, driven by various cytokines and growth factors. These signaling molecules, such as Fibroblast Growth Factor (FGF), Epidermal Growth Factor (EGF), Hepatocyte Growth Factor (HGF), Transforming Growth Factor (TGF)-β, Tumor Necrosis Factor (TNF)-α, and Interleukin-6 (IL-6), are secreted by mesenchymal cells within the septum transversum (Schmidt et al., 1995; Weinstein et al., 2001; Zhao and Duncan, 2005; Tanimizu and Miyajima, 2007). In the liver, hepatoblasts undergoing budding express genes that are characteristic of hepatocytes, including albumin (ALB), transthyretin (TTR), and alpha-fetoprotein (AFP) (Lemaigre and Zaret, 2004; Zaret, 2008). Following invasion of the mesenchyme, these bipotential cells differentiate into hepatocytes (α-fetoprotein^+^/albumin^+^) and cholangiocytes (cytokeratin (CK)-19^+^) (Germain et al., 1988; Jung et al., 1999). The precise equilibrium between hepatocyte and cholangiocyte populations derived from hepatoblasts is maintained through coordinated signaling and transcriptional networks. The Jagged-Notch pathway is crucial in guiding hepatoblast differentiation toward a biliary epithelial phenotype (McCright et al., 2002; Tanimizu and Miyajima, 2004), whereas Hepatocyte Growth Factor (HGF) inhibits biliary differentiation. In conjunction with oncostatin M (OSM), HGF promotes hepatocyte differentiation (Suzuki et al., 2003). After lineage segregation, the proportion of bipotential cells decreases significantly, with most differentiating into either hepatocyte or cholangiocyte lineages. Committed cells undergo progressive morphological and physiological changes, with maturation continuing postnatally for several weeks. This process has been elucidated through various gene array analyses of rodent liver development (Kelley-Loughnane et al., 2002; Jochheim et al., 2003; Petkov et al., 2004).

In this study, human embryonic livers ranging from 2-month-old to 7-month-old were collected for transcriptome sequencing. The study identified differentially expressed genes (DEGs) and biological processes associated with human embryonic liver development at various stages. Our findings establish a foundational basis for investigating liver development in human embryos and identifying diseases caused by abnormalities in embryonic liver function.

## Materials and methods

### Collection of human embryonic livers

This study was approved by the Institutional Review Committee of Rugao Third People’s Hospital and the Affiliated Hospital of Nantong University (Approval No. 2020-K013). All research involving human participants were conducted in strict accordance with the Declaration of Helsinki guidelines. Informed consent was obtained from the participants’ parents before the study commenced. Embryonic liver tissue was collected from legally aborted human fetuses, aged 2–7 months, and stored at −80°C. Only one biological liver sample was collected at each timepoint for analysis, and biological sex of the samples was not determined due to ethical regulations.

### Isolation of liver RNA

Total RNA was isolated using Trizol reagent (Thermo Fisher Scientific, Waltham, MA, USA). RNA concentration and integrity were assessed with a NanoDrop spectrophotometer (Thermo Fisher Scientific) and a 2100 Bioanalyzer (Agilent Technologies, San Diego, CA, United States), respectively.

### RNA sequencing and data analysis

The cDNA libraries, constructed from PCR products, were sequenced using the BGISEQ-500/MGISEQ-2000 system (BGI-Shenzhen, China). Quality control and data cleaning were performed using Fastp. Reference sequence alignment was performed using STAR, and gene counts were obtained through FeatureCounts. Gene ontology (GO) analysis was conducted with the clusterProfiler package. Additionally, gene interaction networks related to lymphocyte differentiation, the regulation of hemopoiesis, and liver development were analyzed and visualized using Cytoscape (Shannon et al., 2003).

### Quantitative real-time PCR assays

Total RNA was extracted from liver tissues, and cDNA was synthesized using a reverse transcription kit. Quantitative real-time PCR (qPCR) analysis was conducted with AceQ^®^ Universal SYBR^®^ qPCR Master Mix (Vazyme Biotech, Nanjing, China) on the LightCycler 96 Real-Time PCR System (Roche, Basel, Switzerland). Primers were acquired from GENEWIZ (Shanghai, China) and were listed in Table 1 mRNA levels were quantified and normalized to β-actin mRNA expression, with fold changes calculated using the 2^−ΔΔCT^ method.

**TABLE 1:** Primers for quantitative RT-PCR.

Genes	Forward primer sequence (5′- 3′)	Reverse primer sequence (5’ - 3′)
CYP26B1	CAT​GCC​CGT​GAC​AGT​GTT​AG	GAG​GAG​GAG​ACG​CTG​AAG​AG
TBX21	GCC​CAC​CAT​GTC​CTA​CTA​CC	CAT​CTT​GGG​AGG​GTA​CTG​GG
LAG3	TGG​AGC​AGC​AGT​GTA​CTT​CA	AGG​AGC​AGA​GAA​AGG​ACA​CC
IL4I1	GCC​CGA​AGA​CAT​CTA​CCA​GA	TTC​CCC​TCC​CCG​AGA​AGA​TA
PTN	ACT​GTC​ACC​ATC​TCC​AAG​CC	TCT​CCT​GTT​TCT​TGC​CTT​CCT
VNN1	GAA​GCC​ATG​CGA​TAC​CAG​TG	CTC​AGG​CTC​CTT​GGG​TAC​AT
LOX	TCA​ATC​CCT​GAA​ATG​TCT​GCC	TGT​GTT​TAG​AGG​TGC​CAG​GA
CD80	ACC​TGG​CTG​AAG​TGA​CGT​TA	GAG​AGG​TGA​GGC​TCT​GGA​AA
PCK1	GTG​GAT​GTT​CAA​CCG​GAT​CG	CTT​CCA​CCT​CCT​TCT​CCC​AG
IRF1	GGT​GAA​CAG​GGA​CAT​GCA​TC	GTC​TCG​AAC​TCC​TGA​CCT​CA
IL2RA	CAA​AGT​CCA​ATG​CAG​CCA​GT	ATA​AAC​CAT​CTG​CCC​CAC​CA
IL10	AAG​CTG​AGA​ACC​AAG​ACC​CA	ACG​GCC​TTG​CTC​TTG​TTT​TC
IRF4	GTC​TGA​ATG​GTG​CGT​GAA​GG	GAG​ATC​CAC​CTG​CAT​CGA​GA
IL15	TAT​GTA​TTG​GTG​GGG​CTG​GG	AGG​AAG​TCA​ATG​AGA​GCC​AGT
TRIB1	ACC​AGG​ACA​AAA​TCA​GGC​CT	GAG​TGC​ATG​TCC​CCA​AAG​TC
TCIM	TCA​TCA​TGT​CCA​CGT​CGC​TA	TTC​AAA​GAT​GTT​GCC​CAC​GG
EGR3	AGG​AGA​AGA​AGG​CGG​AGA​AG	AGG​GGA​AAA​GTG​GGG​ATC​TG
IRF7	CAC​ACA​CAC​ATG​CTG​GAC​TC	CCT​TGG​TTG​GGA​CTG​GAT​CT
ZFP36L1	GGA​TTC​TCT​CTC​GGA​CCA​GG	TCC​CTA​CCC​TGG​CTT​AGT​CA
ISG15	CCT​GAC​GGT​GAA​GAT​GCT​GG	ATC​TTC​TGG​GTG​ATC​TGC​GC
HLA-DRB1	CCT​GAC​GCT​GAG​TAC​TGG​AA	CTC​TCC​ACA​ACC​CCG​TAG​TT
HGF	CTG​GGG​CTA​CAC​TGG​ATT​GA	TTC​AGA​GTC​ACC​TTC​CCT​CG
CPS1	CCA​CAC​AAA​CCA​TCA​GCC​AA	TGG​ACG​TTG​AAT​GGA​CCA​GA
OTC	CCT​TCA​GGC​AGC​TAC​TCC​AA	AAT​ACA​TTG​CCT​CCA​TGC​GC
PROX1	CTC​ATA​TTC​AGA​GCT​GGG​ATC​A	AGG​CTC​AGT​TGA​AAA​GAC​TCA
ASS1	CGT​GCA​GGG​TGA​TTA​TGA​GC	ACG​GGT​CTA​TTT​GGC​AGT​GA
GATA6	TTC​CCA​TGA​CTC​CAA​CTT​CCA	CCT​GAG​GCT​GTA​GGT​TGT​GT
ACTIN	TTG​TTA​CAG​GAA​GTC​CCT​TGC​C	ATG​CTA​TCA​CCT​CCC​CTG​TGT​G

### Western blot

Proteins were extracted from liver tissues using RIPA Lysis Buffer, and the concentrations were quantified using a BCA protein assay kit (Biosharp). Equal volumes of protein samples were separated by sodium dodecyl sulfate-polyacrylamide gel electrophoresis (SDS-PAGE) at 10% concentration, and then transferred to a polyvinylidene fluoride (PVDF) membrane. The membranes were blocked with 5% skim milk for 1–2 h and then incubated overnight at 4°C with primary antibodies specific to PCK1. Subsequently, the membranes were incubated with corresponding secondary antibodies (GAPDH) for 1–2 h at room temperature. Protein bands were visualized using ECL reagent, and the Image Lab software was employed to analyze the expression of the target protein.

### Statistical analysis

The Student’s t-test and one-way analysis of variance (ANOVA) were employed to evaluate the differences between two groups and among multiple groups, respectively. Results were reported as mean ± standard deviation (SD). Statistical analyses were conducted using GraphPad Prism 7.0 (GraphPad Software, Inc., San Diego, CA, United States), with differences considered statistically significant at *p* < 0.05.

## Results

### Analysis of differentially expressed genes and associated biological processes in the liver of 3-month-old human embryo

**FIGURE 1:**
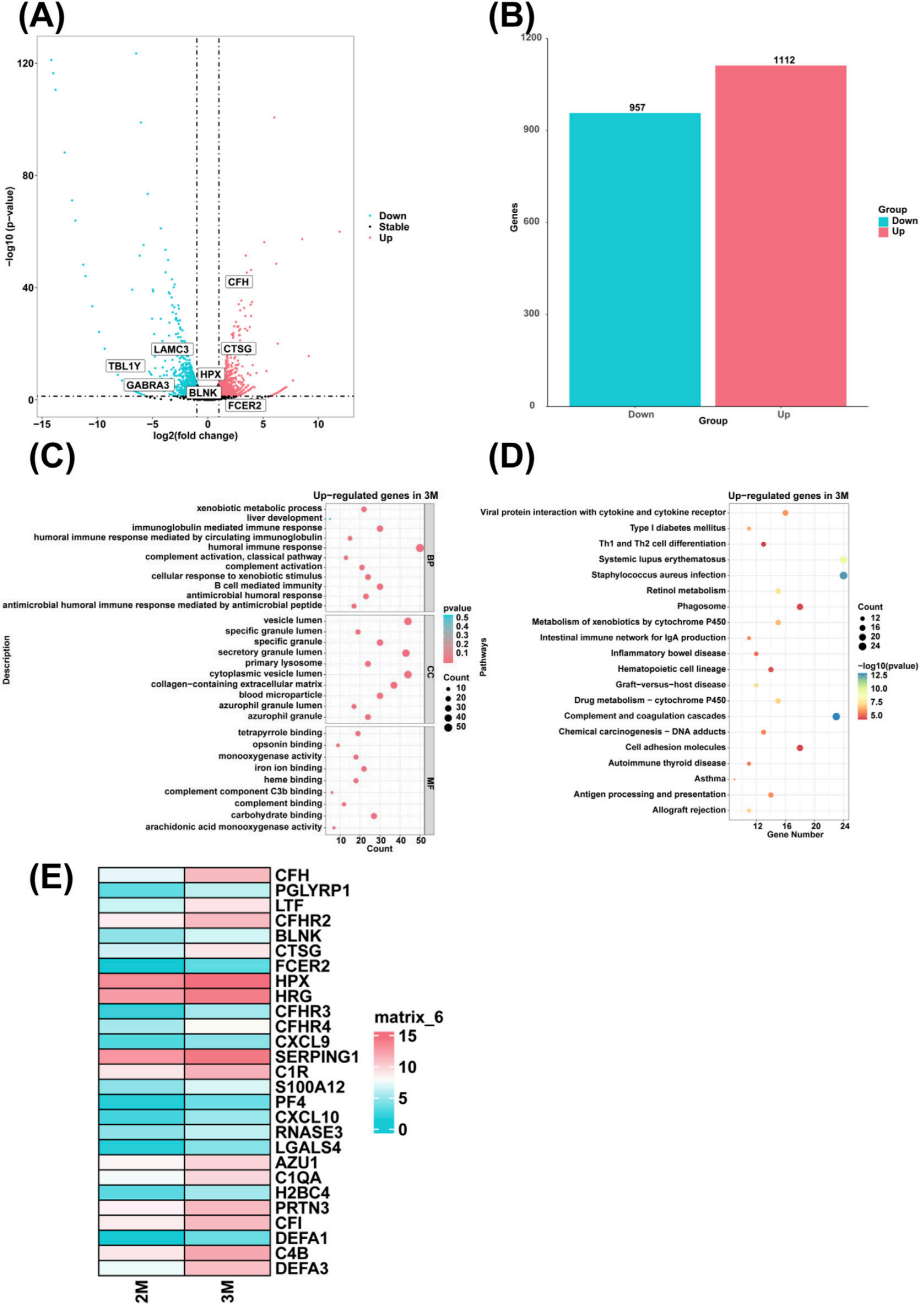
The genes with high expression levels and biological processes in the liver of 3-month-old human embryo. **(A)** The volcano plot showed the differentially expressed gene (DEGs) between the livers of 2-month-old and 3-month-old human embryos. **(B)** A total of 1,112 upregulated and 957 downregulated genes were identified in the liver of 3-month-old human embryo (3-month-old vs. 2-month-old liver). **(C)** GO analysis of the highly expressed genes identified in the liver of 3-month-old human embryo. **(D)** KEGG pathway analysis of the highly expressed genes identified in the liver of 3-month-old human embryo. **(E)** Heatmaps showed the expression of genes associated with humoral immune response in the livers of 2-month-old and 3-month-old human embryos.

To examine genes with high expression in the liver of 3-month-old human embryo, differentially expressed genes (DEGs) between 2-month-old and 3-month-old embryonic liver tissues were identified through RNA sequencing. In comparison to the 2-month-old embryonic liver, 1,112 upregulated genes and 957 downregulated genes were identified in the liver of 3-month-old human embryo (Figures 1A, B). To elucidate the biological function of DEGs, GO analysis revealed that highly expressed genes in the 3-month-old embryonic liver were enriched in the biological processes such as humoral immune response, complement activation, xenobiotic metabolic process, antimicrobial humoral response, immunoglobulin mediated immune response, complement activation, classical pathway, B cell mediated immunity, humoral immune response mediated by circulating immunoglobulin, antimicrobial humoral immune response mediated by antimicrobial peptide, cellular response to xenobiotic stimulus, and liver development (Figure 1C). KEGG pathway analysis showed that the highly expressed genes in the 3-month-old embryonic liver were associated with hematopoietic cell lineage, complement and coagulation cascades (Figure 1D). Compared to the 2-month-old embryonic liver, heatmaps revealed that genes associated with the biological process of humoral immune response, such as *CFH*, *PGLYRP1*, *LTF*, *CFHR2*, *BLNK*, *CTSG*, *FCER2*, *HPX*, *HRG*, *CFHR3*, *CFHR4*, *CXCL9*, *SERPING1*, *C1R*, *S100A12*, *PF4*, *CXCL10*, *RNASE3*, *LGALS4*, *AZU1*, *C1QA*, *H2BC4*, *PRTN3*, *CFI*, *DEFA1*, *C4B*, *DEFA3* were highly expressed in the liver of the 3-month-old human embryo (Figure 1E).

### Analysis of differentially expressed genes and associated biological processes in the liver of 4-month-old human embryo

**FIGURE 2:**
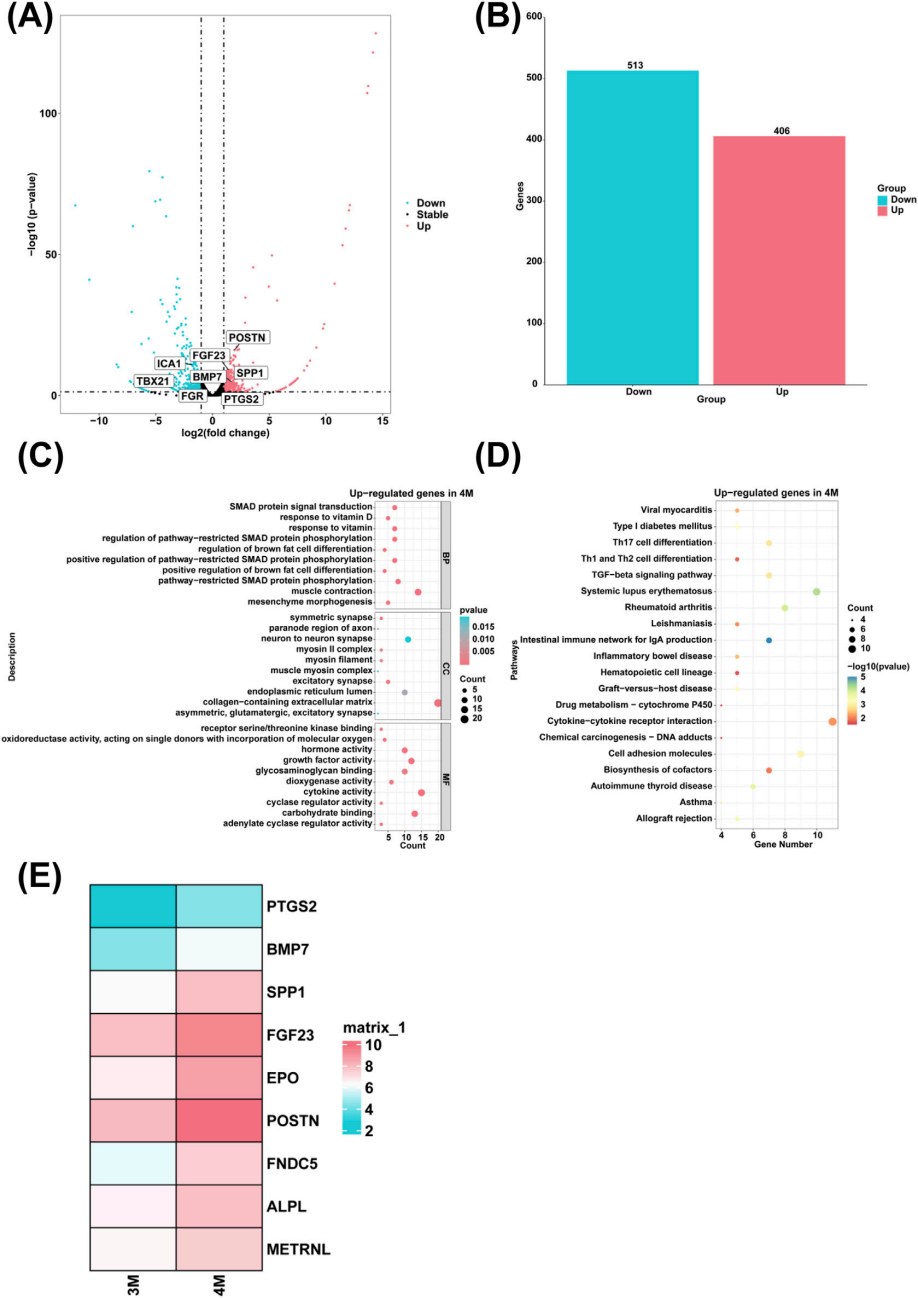
The genes with high expression levels and biological processes in the liver of 4-month-old human embryo. **(A)** The volcano plot showed the DEGs between the livers of 3-month-old and 4-month-old human embryos. **(B)** A total of 406 upregulated genes and 513 downregulated genes were identified in the liver of 4-month-old human embryo (4-month-old vs. 3-month-old liver). **(C)** GO analysis of the highly expressed genes identified in the liver of 4-month-old human embryo. **(D)** KEGG pathway analysis of the highly expressed genes identified in the liver of 4-month-old human embryo. **(E)** Heatmaps showed the expression of genes associated with response to vitamin and brown fat cell differentiation in the livers of 3-month-old and 4-month-old human embryos.

To investigate genes with high expression levels in the liver of 4-month-old human embryo, we identified differentially expressed genes (DEGs) between the 3-month-old and 4-month-old embryonic liver using RNA sequencing. Compared to the 3-month-old embryonic liver, 406 upregulated genes and 513 downregulated genes were identified in the liver of 4-month-old human embryo (Figures 2A, B). To explore the biological functions of these DEGs, GO analysis revealed that highly expressed genes in the 4-month-old embryonic liver were enriched in the biological processes such as response to vitamin D, response to vitamin, positive regulation of brown fat cell differentiation, regulation of brown fat cell differentiation, pathway-restricted SMAD protein phosphorylation, positive regulation of pathway-restricted SMAD protein phosphorylation, regulation of pathway-restricted SMAD protein phosphorylation, SMAD protein signal transduction, mesenchyme morphogenesis and muscle contraction (Figure 2C). KEGG pathway analysis showed that highly expressed genes in the 4-month-old embryonic liver were associated with signaling pathways like Th17 cell differentiation and allograft rejection (Figure 2D). Compared to the 3-month-old embryonic liver, heatmap analysis further demonstrated that genes associated with the biological process of response to vitamin and brown fat cell differentiation, such as *PTGS2*, *BMP7*, *SPP1*, *FGF23*, *EPO*, *POSTN*, *ALPL*, *FNDC5*, *METRNL* were highly expressed in the liver of 4-month-old human embryo (Figure 2E).

### Analysis of differentially expressed genes and associated biological processes in the liver of 5-month-old human embryo

**FIGURE 3:**
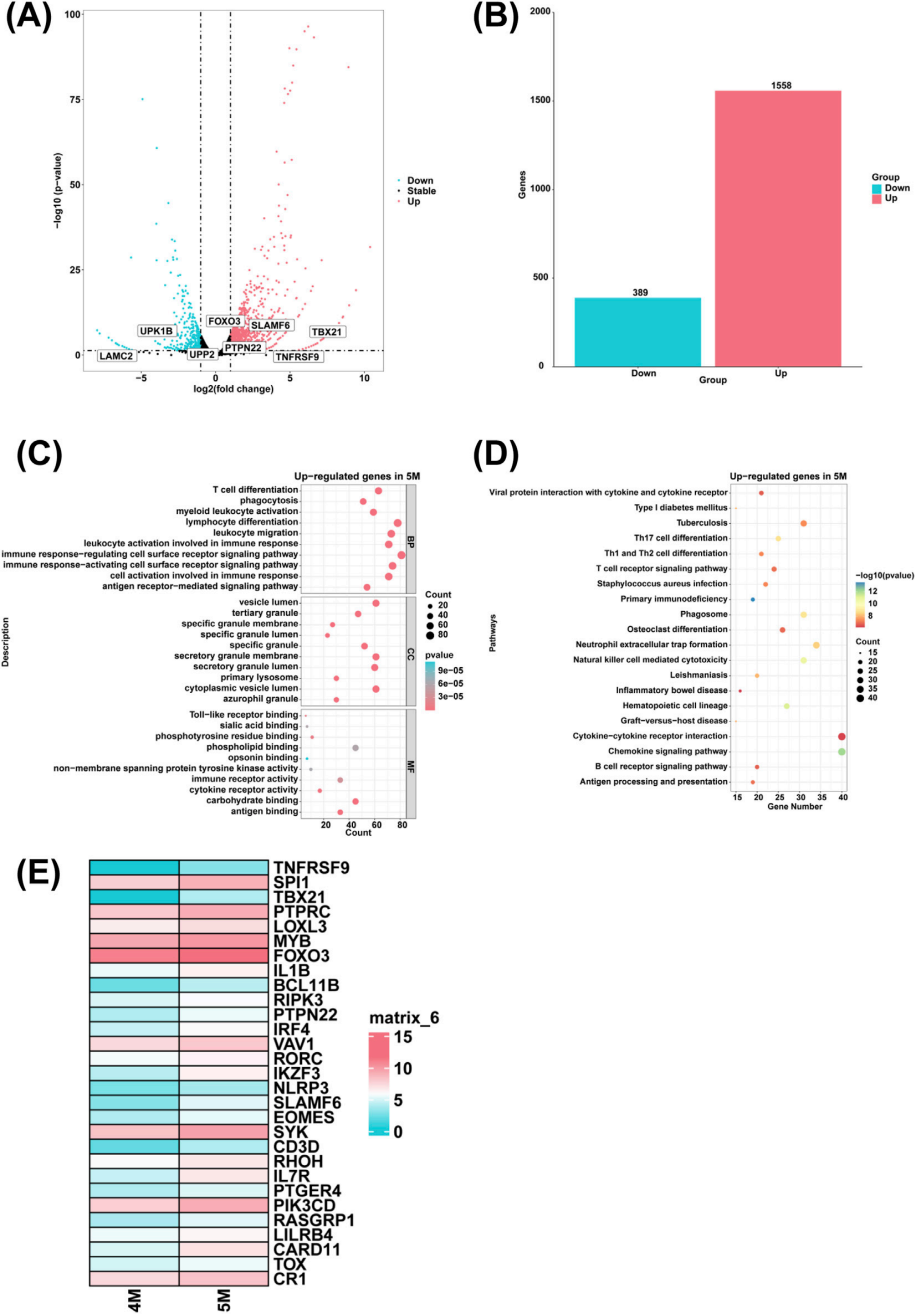
The genes with high expression levels and biological processes in the liver of 5-month-old human embryo. **(A)** The volcano plot showed the DEGs between the livers of 4-month-old and 5-month-old human embryos. **(B)** A total of 1,558 upregulated genes and 389 downregulated genes were identified in the liver of 5-month-old human embryo (5-month-old vs. 4-month-old liver). **(C)** GO analysis of the highly expressed genes identified in the liver of 5-month-old human embryo. **(D)** KEGG pathway analysis of the highly expressed genes identified in the liver of 5-month-old human embryo. **(E)** Heatmaps showed the expression of genes associated with T cell differentiation in the livers of 5-month-old and 6-month-old human embryos.

To investigate the genes with high expression in the liver of the 5-month-old human embryo, differentially expressed genes (DEGs) between the 4-month-old and 5-month-old embryonic liver were identified via RNA sequencing. Compared to the 4-month-old embryonic liver, 1,558 upregulated genes and 389 downregulated genes were identified in the liver of 5-month-old human embryo (Figures 3A, B). To explore the biological function of DEGs, GO analysis indicated that highly expressed genes of the 5-month-old embryonic liver were enriched in the biological processes such as T cell differentiation, phagocytosis, myeloid leukocyte activation, leukocyte migration, leukocyte activation involved in immune response, immune response-activating cell surface receptor signaling pathway, immune response-regulating cell surface receptor signaling pathway, cell activation involved in immune response, antigen receptor-mediated signaling pathway, and lymphocyte differentiation (Figure 3C). Additionally, KEGG pathway analysis revealed that highly expressed genes of the 5-month-old embryonic liver were associated with the signaling pathways like primary immunodeficiency, phagosome and hematopoietic cell lineage (Figure 3D). Compared to the 4-month-old embryonic liver, heatmaps showed that genes associated with T cell differentiation, such as *TNFRSF9*, *SPI1*, *TBX21*, *PTPRC*, *LOXL3*, *MYB*, *FOXO3*, *IL1B*, *BCL11B*, *RIPK3*, *PTPN22*, *IRF4*, *VAV1*, *RORC*, *IKZF3*, *NLRP3*, *SLAMF6*, *EOMES*, *SYK*, *CD3D*, *RHOH*, *IL7R*, *PTGER4*, *PIK3CD*, *RASGRP1*, *LILRB4*, *CARD11*, *TOX*, *CR1* were highly expressed in the liver of 5-month-old human embryo (Figure 3E).

### Analysis of differentially expressed genes and associated biological processes in the liver of 6-month-old human embryo

**FIGURE 4:**
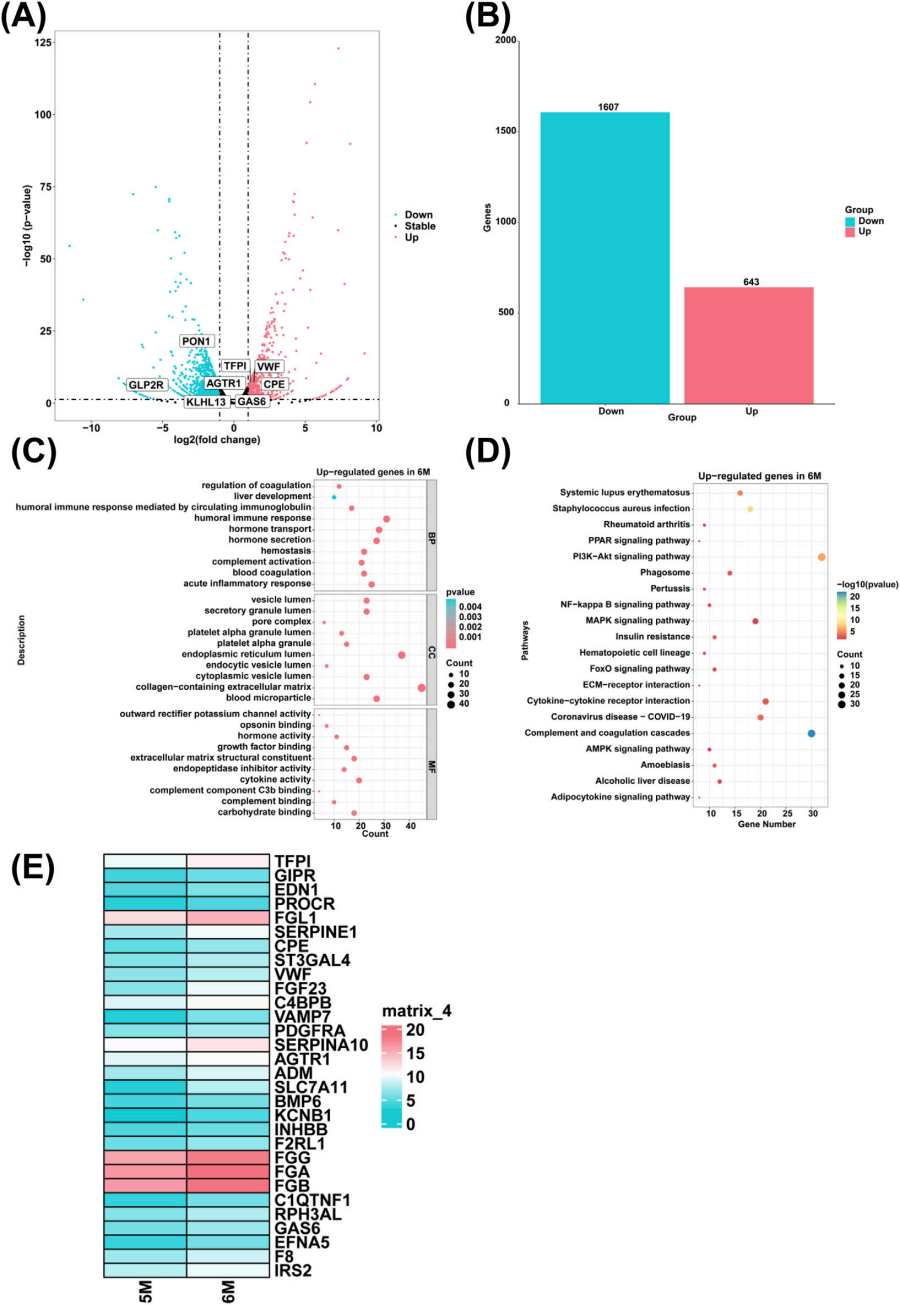
The genes with high expression levels and biological processes in the liver of 6-month-old human embryo. **(A)** The volcano plot showed the DEGs between the livers of 5-month-old and 6-month-old human embryos. **(B)** A total of 643 upregulated genes and 1,607 downregulated genes were identified in the liver of 6-month-old human embryo (6-month-old vs. 5-month-old liver). **(C)** GO analysis of the highly expressed genes identified in the liver of 6-month-old human embryo. **(D)** KEGG pathway analysis of the highly expressed genes identified in the liver of 6-month-old human embryo. **(E)** Heatmaps showed the expression of genes associated with coagulation and hormone transport in the livers of 5-month-old and 6-month-old human embryos.

To investigate genes with high expression levels in the liver of 6-month-old human embryo, we identified differentially expressed genes (DEGs) between the 5-month-old and the 6-month-old embryonic liver through RNA sequencing. Compared to the 5-month-old embryonic liver, 643 upregulated genes and 1,607 downregulated genes were determined in the liver of 6-month-old human embryo (Figures 4A, B). GO analysis showed that highly expressed genes of the 6-month-old embryonic liver were enriched in the biological processes such as the regulation of coagulation, blood coagulation, complement activation, acute inflammatory response, liver development, humoral immune response mediated by circulating immunoglobulin, humoral immune response, hormone transport, hormone secretion, hemostasis (Figure 4C). KEGG pathway analysis revealed that highly expressed genes in the 6-month-old embryonic liver were associated with the signal pathways like complement and coagulation cascades, amoebiasis, alcoholic liver disease (Figure 4D). Compared to the 5-month-old embryonic liver, heatmap analysis showed that the genes associated with the biological process of coagulation and hormone transport, such as *TFPI*, *VWF*, *FGG*, *FGA*, *FGB*, *GAS6*, *F8*, *GIPR*, *CPE*, *FGF23*, *VAMP7*, *AGTR1*, *ADM*, *BMP6*, *KCNB1*, *RPH3AL*, *EFNA5*, *IRS2*, *EDN1*, *PROCR*, *FGL1*, *SERPINE1*, *ST3GAL4*, *INHBB, F2RL1*, *C1QTNF1*, *C4BPB*, *PDGFRA*, *SERPINA10*, *SLC7A11*, *F2RL1* were highly expressed in the liver of 6-month-old human embryo (Figure 4E).

### Analysis of differentially expressed genes and associated biological processes in the liver of 7-month-old human embryo

**FIGURE 5:**
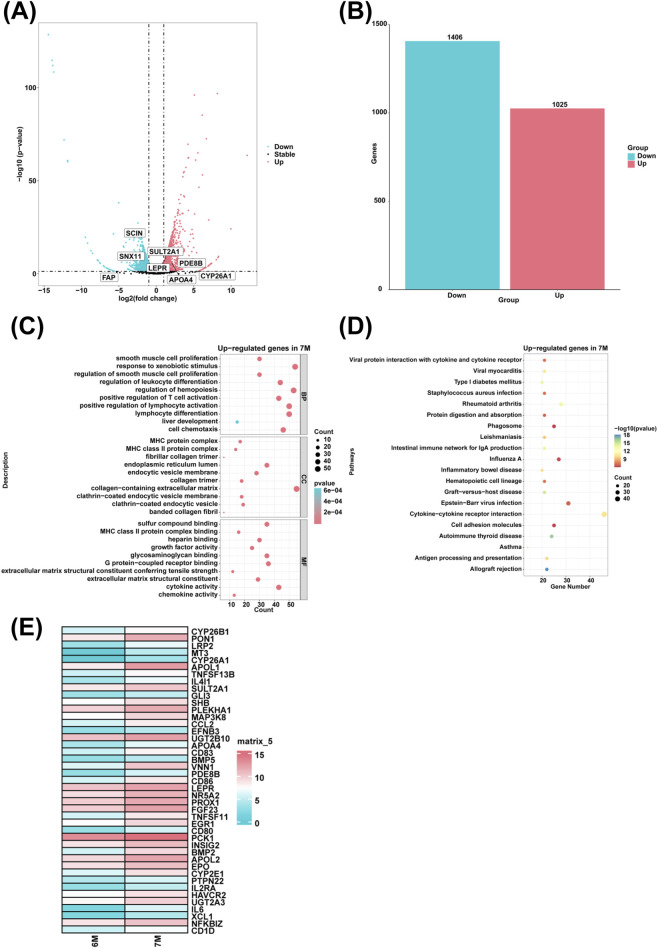
The genes with high expression and biological processes in the liver of 7-month-old human embryo. **(A)** The volcano plot showed the DEGs between the livers of 6-month-old and 7-month-old human embryos. **(B)** A total of 1,025 upregulated and 1,406 downregulated genes were identified in the liver of 7-month-old human embryo (7-month-old vs. 6-month-old liver). **(C)** GO analysis of the highly expressed genes identified in the 7-month-old human embryonic liver. **(D)** KEGG pathway analysis of the highly expressed genes identified in the 7-month-old embryonic liver. **(E)** Heatmaps showed the expression of genes associated with positive regulation of T cell activation and steroid metabolic process in the livers of 6-month-old and 7-month-old human embryos.

To investigate genes with high expression in the liver of 7-month-old human embryo, differentially expressed genes (DEGs) between the 6-month-old and 7-month-old embryonic liver were identified through RNA sequencing. Compared to the 6-month-old embryonic liver, 1,025 upregulated genes and 1,406 downregulated genes were identified in the liver of 7-month-old human embryo (Figures 5A, B). GO analysis showed that highly expressed genes in the 7-month-old embryonic liver were enriched in the biological processes such as smooth muscle cell proliferation, response to xenobiotic stimulus, regulation of smooth muscle cell proliferation, regulation of leukocyte differentiation, positive regulation of T cell activation, positive regulation of lymphocyte activation, lymphocyte differentiation, liver development, cell chemotaxis, regulation of hemopoiesis (Figure 5C). KEGG pathway analysis indicated that the highly expressed genes in the 7-month-old embryonic liver were associated with signaling pathways involved in allograft rejection and graft-versus-host disease (Figure 5D). Compared to the 6-month-old embryonic liver, heatmap analysis showed that the genes associated with the biological process of positive regulation of T cell activation and steroid metabolic process, such as *TNFSF13B*, *IL4I1*, *GLI3*, *SHB*, *MAP3K8*, *CCL2*, *EFNB3*, *CD83*, *VNN1*, *CD86*, *TNFSF11*, *CD80*, *PCK1*, *EPO*, *PTPN22, IL2RA*, *HAVCR2*, *IL6*, *XCL1*, *NFKBIZ*, *CD1D*, *CYP26B1*, *PON1*, *LRP2*, *MT3*, *CYP26A1*, *APOL1*, *SULT2A1*, *PLEKHA1*, *UGT2B10*, *APOA4*, *BMP5*, *PDE8B*, *LEPR, NR5A2*, *PROX1*, *FGF23*, *EGR1*, *INSIG2*, *BMP2*, *APOL2*, *CYP2E1*, *UGT2A3* were highly expressed in the liver of 7-month-old human embryo (Figure 5E).

### Analysis of gene expression and associated biological processes in the liver along the pseudotime from 3-month-old to 7-month-old

**FIGURE 6:**
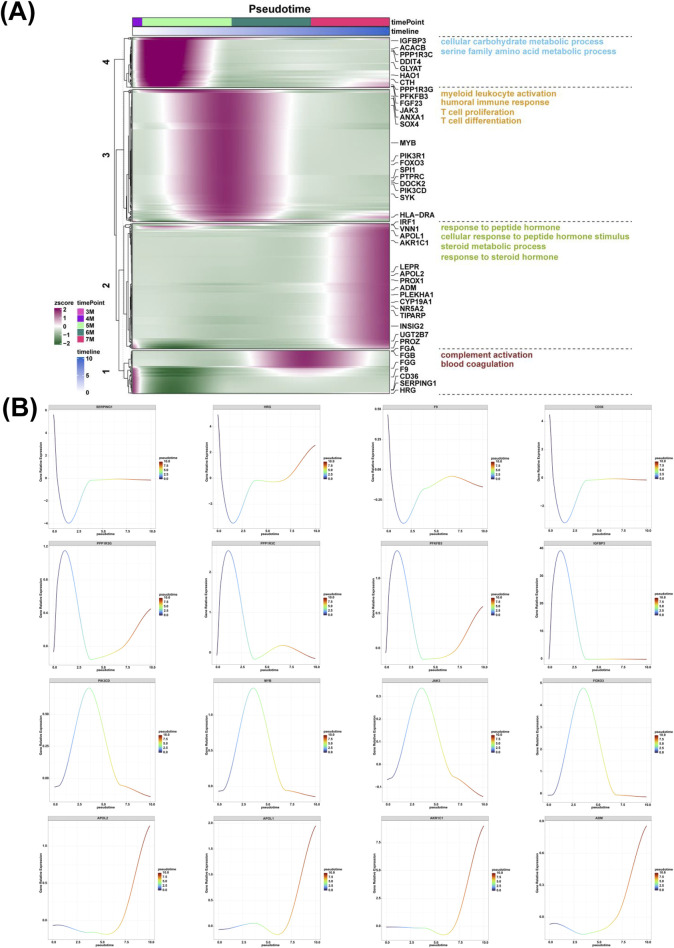
The expression analysis of genes and associated biological processes along the pseudotime from the 3-month-old to 7-month-old liver. **(A)** The biological processes of complement activation and blood coagulation along the pseudotime from the 3-month-old to 4-month-old liver, cellular carbohydrate metabolic process and serine family amino acid metabolic process along the pseudotime from the 4-month-old to 5-month-old liver, myeloid leukocyte activation, humoral immune response, T cell proliferation and T cell differentiation along the pseudotime from the 5-month-old to 6-month-old liver, response to peptide hormone, cellular response to peptide hormone stimulus, steroid metabolic process and response to steroid hormone along the pseudotime of the 7-month-old liver, respectively. **(B)** The expression of *SERPING1*, *HRG*, *F9*, *CD36*, *PPP1R3G*, *PPP1R3C*, *PFKFB3*, *IGFBP3*, *PIK3CD*, *MYB*, *JAK3*, *FOXO3*, *APOL2*, *APOL1*, *AKR1C1*, *ADM* along the pseudotime from the 3-month-old to 7-month-old liver.

The high expression genes along the pseudotime from the 3-month-old to 4-month-old embryonic liver were enriched in the biological process of complement activation and blood coagulation (Figure 6A). The high expression levels of the genes including *SERPING1*, *HRG*, *F9*, *CD36*, *PROZ*, *FGG* were identified (Figure 6B; Supplementary Figure S1A). The high expression genes along the pseudotime from the 4-month-old to 5-month-old embryonic liver were enriched in the biological process of cellular carbohydrate metabolic process and serine family amino acid metabolic process (Figure 6A). The high expression levels of the genes including *PPP1R3G*, *PPP1R3C*, *PFKFB3*, *IGFBP3, DDIT4*, and *ACACB* were determined (Figure 6B; Supplementary Figure S1B). The high expression genes along the pseudotime from the 5-month-old to 6-month-old embryonic liver were enriched in the biological process of myeloid leukocyte activation, humoral immune response, T cell proliferation and T cell differentiation (Figure 6A). The expression of genes such as *PIK3CD*, *MYB*, *JAK3*, *FOXO3*, *SYK*, *PIK3R1*, *SPI1*, *PTPRC*, *SOX4*, *DOCK2*, *ANXA1* were investigated (Figure 6B; Supplementary Figure S1C). The high expression genes along the pseudotime of the 7-month-old embryonic liver were enriched in the biological process of response to peptide hormone, cellular response to peptide hormone stimulus, steroid metabolic process and response to steroid hormone (Figure 6A). The expression of genes including *APOL2*, *APOL1*, *AKR1C1*, *ADM*, *UGT2B7*, *TIPARP*, *PROX1*, *PLEKHA1*, *LEPR*, *INSIG2*, *CYP19A1*, *NR5A2* were also identified (Figure 6B; Supplementary Figure S1D).

### Verification of the expression of genes associated with lymphocyte differentiation, regulation of hemopoiesis and liver development

**FIGURE 7:**
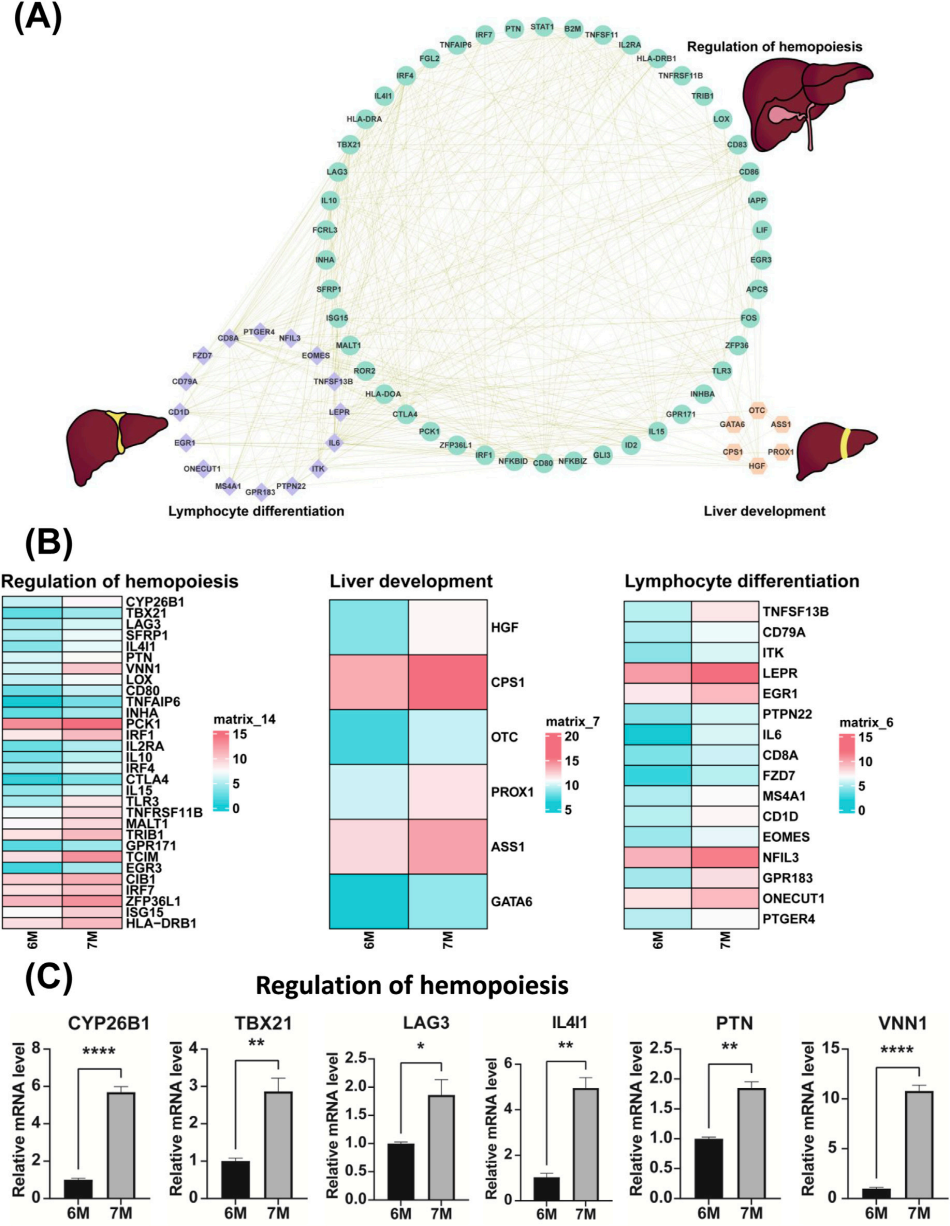
Validation of the genes associated with the biological processes of the regulation of hemopoiesis, liver development and lymphocyte differentiation. **(A)** Potential gene interaction networks involved in the regulation of hemopoiesis, liver development and lymphocyte differentiation. **(B)** Heatmaps illustrate the expression profiles of genes related to the regulation of hemopoiesis, liver development and lymphocyte differentiation between the livers of 6-month-old and 7-month-old human embryos. **(C)** The expression of genes involved in the regulation of hemopoiesis in the livers of 6-month-old and 7-month-old human embryos using qPCR. *p < 0.05, **p < 0.01, ***p < 0.001 and ****p < 0.0001.

**FIGURE 8:**
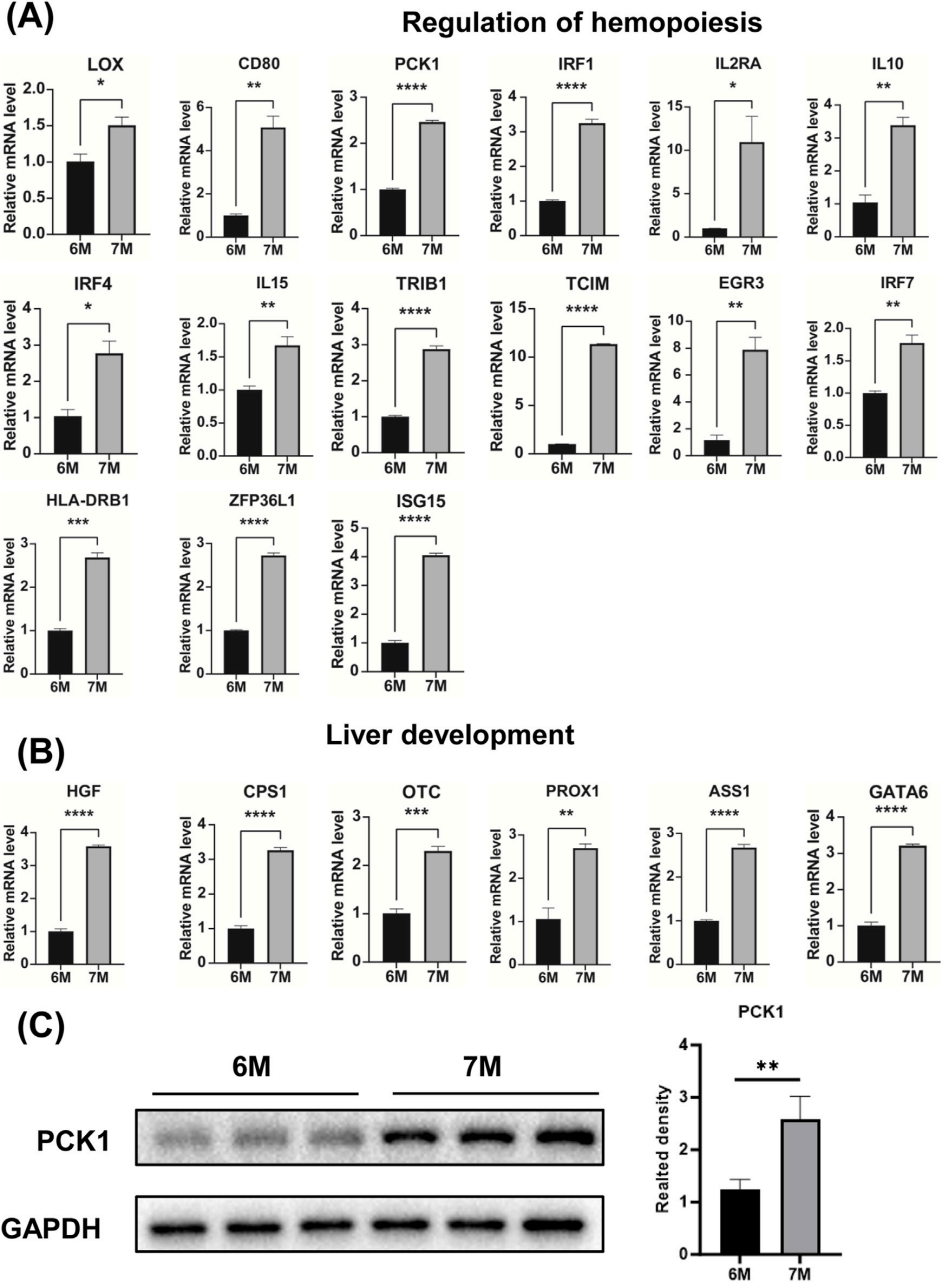
The expression analysis of genes involved in the regulation of hemopoiesis and liver development. **(A)** The expression of genes associated with the regulation of hemopoiesis in the livers of 6-month-old and 7-month-old human embryos using qPCR. **(B)** The expression of genes associated with liver development in the livers of 6-month-old and 7-month-old human embryo using qPCR. **(C)** The expression level of PCK1 were quantified through Western blot analysis and the statistical analysis of protein level of PCK1. *p < 0.05, **p < 0.01, ***p < 0.001 and ****p < 0.0001.

Through the comparative transcriptome sequencing analysis of the liver of 6-month-old and 7-month-old human embryo, we identified the differentially expressed genes (DEGs) associated with lymphocyte differentiation, regulation of hemopoiesis and liver development. The interactive network of genes related to lymphocyte differentiation, regulation of hemopoiesis and liver development were illustrated in Figure 7A. In comparison to the liver of the 6-month-old embryo, the significant upregulation of genes involved in lymphocyte differentiation, regulation of hemopoiesis and liver development were observed in the liver of 7-month-old human embryo. As illustrated in Figure 7B, these genes include *CYP26B1*, *TBX21*, *LAG3*, *SFRP1*, *IL4I1*, *PTN*, *VNN1*, *LOX*, *CD80*, *TNFAIP6*, *INHA*, *PCK1*, *IRF1*, *IL2RA*, *IL10*, *IRF4*, *CTLA4*, *IL15*, *TLR3*, *TNFRSF11B*, *MALT1*, *TRIB1*, *GPR171*, *TCIM*, *EGR3*, *CIB1*, *IRF7*, *ZFP36L1*, *ISG15*, *HLA-DRB1*, *HGF*, *CPS1*, *OTC*, *PROX1*, *ASS1*, *GATA6*, *TNFSF13B*, *CD79A*, *ITK*, *LEPR*, *EGR1*, *PTPN22*, *IL6*, *CD8A*, *FZD7*, *MS4A1*, *CD1D*, *EOMES*, *NFIL3*, *GPR183*, *ONECUT1*, *PTGER4*. The expression of these genes in other developmental stages was also investigated (Supplementary Figure S3). Quantitative PCR (qPCR) analysis confirmed the expression of these genes, with results consistent with the RNA sequencing data (Figures 7C, 8A). The expression validation of the PCK1 protein was conducted in this study (Figure 8C).

### Analysis of the low expression genes and their associated biological processes in the liver at different developmental stages

To investigate genes with low expression in the liver of various developmental stages, downregulated genes were identified using RNA sequencing. In comparison to the liver of 2-month-old human embryo, GO analysis revealed that the downregulated genes in the liver of 3-month-old human embryo were involved in renal system development and kidney development (Supplementary Figure S2A). KEGG pathway analysis identified the alcoholism signaling pathway among the downregulated genes in the 3-month-old embryonic liver (Supplementary Figure S2B). Comparative analysis of the livers from 3-month-old and 4-month-old human embryos indicated that the downregulated genes in the liver of 4-month-old human embryo were associated with the biological processes of humoral immune response and leukocyte chemotaxis (Supplementary Figure S2C). KEGG pathway analysis identified the complement and coagulation cascade signaling pathways among the downregulated genes in the liver of 4-month-old human embryo (Supplementary Figure S2D). GO analysis revealed that the downregulated genes in the liver of 5-month-old human embryo were involved in the biological processes of mesenchymal cell differentiation, gland morphogenesis, and mesenchyme development (Supplementary Figure S2E). KEGG pathway analysis indicated that downregulated genes in the liver of 5-month-old embryo were associated with mineral absorption and axon guidance signaling pathways (Supplementary Figure S2F). GO analysis demonstrated that the downregulated genes in the liver of 6-month-old human embryo were involved in alcohol metabolic process and xenobiotic metabolic process (Supplementary Figure S2G). KEGG pathway analysis further revealed the signaling pathways related to the metabolism of xenobiotics by cytochrome P450 and cell adhesion molecules among the downregulated genes in the liver of 6-month-old embryo (Supplementary Figure S2H). GO analysis indicated that the downregulated genes in the liver of 7-month-old human embryo were involved in the biological processes of exocytosis and regulation of regulated secretory pathway (Supplementary Figure S2I). KEGG pathway analysis identified the chemokine signaling pathway, calcium signaling pathway and efferocytosis among the downregulated genes in the liver of 7-month-old human embryo (Supplementary Figure S2J).

## Discussion

The normal development of the liver during embryogenesis is crucial to the proper function of the adult liver. To explore the predominantly expressed genes and associated biological processes at various developmental stages of embryonic liver, we collected human embryonic liver tissue ranging from 2-month-old to 7-month-old. We then employed RNA sequencing analysis to identify differentially expressed genes (DEGs). Our research provides researchers with transcriptomic profiles of normal embryonic liver development, validates the expression of essential genes in this process, and offers a reference for studies on embryonic abortion resulting from abnormal liver development.

Compared to the liver of 2-month-old human embryo, the upregulated genes associated with humoral immune response and B cell mediated immunity were identified in the liver of 3-month-old human embryo (Figure 1C). The genes, such as *CFH*, *PGLYRP1*, *LTF*, *CFHR2*, *BLNK*, *CTSG*, *FCER2*, *HPX*, *HRG*, *CFHR3*, *CFHR4*, *CXCL9*, *SERPING1*, *C1R*, *S100A12*, *PF4*, *CXCL10*, *RNASE3*, *LGALS4*, *AZU1*, *C1QA*, *H2BC4*, *PRTN3*, *CFI*, *DEFA1*, *C4B*, *DEFA3* were highly expressed in the liver of 3-month-old human embryo, indicating a distinct immune profile at this development stage (Figure 1E). Previous research suggested that complement factor H (CFH) regulated alternative complement activation by inhibiting the cleavage of the central complement component C3 (Kiss et al., 2019). Lactoferrin (LTF), a critical molecule in human first-line defense against infections, is frequently targeted by humoral autoimmune responses (Hu et al., 2017). The B-cell linker protein (BLNK), also known as BASH or SLP-65, encodes an adaptor protein essential for B-cell receptor signaling (Kurata et al., 2021). Heparin interacts with the endogenous tetrameric protein platelet factor 4 (PF4), forming PF4/heparin complexes that can cause a severe immune-mediated adverse reaction called heparin-induced thrombocytopenia (HIT) (Nguyen et al., 2015). The upregulation of these genes in the liver of 3-month-old human embryo underscores the dominance of humoral responses mediated by B cells as primary biological processes during this developmental stage. This suggests an early establishment of immune functions crucial for fetal protection. The prominence of B-cell-mediated responses in the embryonic liver likely plays a crucial role in the maturation of the immune system.

Compared to the liver of 3-month-old human embryo, the upregulated genes related to the biological process of vitamin response and regulation of brown fat cell differentiation, such as *PTGS2*, *BMP7*, *SPP1*, *FGF23*, *EPO*, *POSTN*, *ALPL*, *FNDC5*, *METRNL* were identified in the liver of 4-month-old human embryo (Figure 2E). Previous studies have demonstrated that BMP7 induces the conversion of primary human adipose stem cells from white to brown (Elsen et al., 2014). Bone morphogenetic protein 7 (BMP7) plays a critical role in the differentiation and development of brown adipose tissue (BAT) (Syamsunarno et al., 2021). Additionally, BMP7 contains a vitamin D receptor (VDR) response element within its promoter region and contributes to cellular response to mechanical loading (Santos et al., 2011). Vitamin D exerts its influence on viral hepatitis through non-genomic factors, including matrix metalloproteinase, endothelial vascular growth factor, prostaglandins, PTGS2 (Cyclooxygenase-2), and oxidative stress (Luong and Nguyen, 2012). Cyclooxygenase-2 regulates energy homeostasis in mice by recruiting brown adipocytes (Vegiopoulos et al., 2010). Vitamin C protects retinal ganglion cells by upregulating SPP1 expression in glaucoma (Li and Jakobs, 2023). Fibroblast growth factor 23 (FGF23) is a critical regulator of both phosphate and vitamin D homeostasis. It participates in an FGF23-Vitamin D-PTH (parathyroid hormone) regulatory axis that governs mineral homeostasis (Blau and Collins, 2015). Periostin (POSTN), a member of a newly identified family of vitamin K-dependent proteins, is expressed by mesenchymal stromal cells (Coutu et al., 2008). Alkaline phosphatase (ALPL) is an essential enzyme that influences the bioavailability of vitamin B6, functioning as a rate-limiting factor in its metabolism (Spinelli et al., 2023). FNDC5, a membrane protein, undergoes cleavage and is secreted as irisin. Irisin acts on white adipose cells to stimulate the expression of thermogenin (UCP1), inducing a program of brown fat-like development (Bostrom et al., 2012). Meteorin-like (METRNL), a hormone secreted by various tissues, including thermogenically active brown and beige adipose tissues, is associated with brown adipose tissue activity in early infancy (Garcia-Beltran et al., 2023). The upregulation of these genes indicated that the 4-month-old embryonic liver is actively engaged in vitamin response and brown fat cell differentiation regulation. This indicates that the liver in the 4-month-old human embryo is actively involved in essential metabolic processes, including nutrient sensing and thermogenesis. The regulatory mechanisms at this stage may be critical for maintaining proper energy homeostasis and could have enduring effects on the health of the human embryos.

In comparison to the liver of 4-month-old human embryo, the upregulated genes associated with the biological process of T cell differentiation, such as *TNFRSF9*, *SPI1*, *TBX21*, *PTPRC*, *LOXL3*, *MYB*, *FOXO3*, *IL1B*, *BCL11B*, *RIPK3*, *PTPN22*, *IRF4*, *VAV1*, *RORC*, *IKZF3*, *NLRP3*, *SLAMF6*, *EOMES*, *SYK*, *CD3D*, *RHOH*, *IL7R*, *PTGER4*, *PIK3CD*, *RASGRP1*, *LILRB4*, *CARD11*, *TOX*, *CR1* were identified in the liver of 5-month-old human embryo (Figure 3E). CD137, also known as TNFRSF9, plays a critical role in the activation of T cells and NK cells, as well as cytokine production (Vinay et al., 2004). The T cell-specific T-box transcription factor TBX21 is integral to immune system regulation, promoting the differentiation of T helper 1 (T(H)1) cells while inhibiting the commitment of T helper 2 (T(H)2) cells, in conjunction with the homeobox transcription factor HLX1 (Suttner et al., 2009). PTPRC enhances immune responses mediated by CD8^+^ T cells and increases drug sensitivity in breast cancer treatment (Li et al., 2023). FOXO3 regulates the memory of CD8^+^ T cells through intrinsic cellular mechanisms (Sullivan et al., 2012). BCL11B is essential for T-cell development and the preservation of T-cell identity (Liu et al., 2010). RIPK3 and Caspase-1/11 are required for the optimal antigen-specific CD8^+^ T cell response (Rana et al., 2020). PTPN22 associates with end-binding protein 1 (EB1) to modulate T-cell receptor signaling (Zhang et al., 2020). SLAMF6 is crucial for T cell activation, enhancing T cell functionality (Gartshteyn et al., 2023). CD3D is responsible for initiating T cell signal transduction and is associated with the antitumor immune response across various cancer types (Wei et al., 2022). Concurrent deficiencies in PIK3CD and TNFRSF9 result in chronic active Epstein-Barr virus infections in T cells (Rodriguez et al., 2019). LILRB4 signaling in leukemia cells promotes T cell suppression and tumor infiltration (Deng et al., 2018). CARD11 signaling is vital for regulating T cell development and function (Deng et al., 2018). Furthermore, TOX plays a critical role in the differentiation of tumor-specific T cells (Scott et al., 2019). The increased expression of these genes in the liver of 5-month-old human embryo indicates significant biological processes, primarily involving the immune system, are actively occurring. These processes encompass T cell differentiation, which is essential for adaptive immune responses, and myeloid leukocyte activation, crucial for innate defense mechanisms. Additionally, lymphocyte differentiation reflects the maturation of B cells and T cells, which are necessary for antigen-specific immunity. Collectively, these biological processes suggest an advanced developmental stage of the embryonic immune system.

In comparison to the liver of 5-month-old human embryo, the upregulated genes associated with coagulation regulation, hemostasis, hormone transport, hormone secretion, and blood coagulation, such as *TFPI*, *VWF*, *FGG*, *FGA*, *FGB*, *GAS6*, *F8*, *GIPR*, *CPE*, *FGF23*, *VAMP7*, *AGTR1*, *ADM*, *BMP6*, *KCNB1*, *RPH3AL*, *EFNA5*, *IRS2*, *EDN1*, *PROCR*, *FGL1*, *SERPINE1*, *ST3GAL4*, *INHBB*, *F2RL1*, *C1QTNF1*, *C4BPB*, *PDGFRA*, *SERPINA10*, *SLC7A11*, *F2RL1* were identified in the liver of 6-month-old human embryo (Figure 4E). The suppression of Tissue Factor Pathway Inhibitor (TFPI) activity can re-establish effective hemostasis via the extrinsic blood coagulation pathway, independently of factors VIII or IX (Peterson et al., 2016). Fibrinogen, a hexameric glycoprotein encoded by three clustered genes including FGA, FGB, and FGG on chromosome 4q, plays a critical role in the final stages of coagulation as a precursor to fibrin monomers (Acharya and Dimichele, 2008). The growth arrest-specific 6 (*GAS6*) gene and its receptor, *AXL*, are pivotal in vascular hemostasis and the pathogenesis of atherosclerosis (Lee et al., 2014). The von Willebrand factor (VWF) is a critical glycoprotein that facilitates primary hemostasis (Kanaji et al., 2012). The gastrointestinal peptide hormone receptor GIPR is involved in linking energy availability to the regulation of hematopoiesis (Pujadas et al., 2020). Carboxypeptidase E (CPE) plays a vital role in processing prohormones into mature hormones and is abundantly expressed in various neuroendocrine tissues (Chen et al., 2023). Fibroblast Growth Factor 23 (FGF23) is a phosphaturic hormone that originates from bone and regulates phosphate and vitamin D metabolism (Takashi and Fukumoto, 2018). The effects of Angiotensin II (ANG II) on blood pressure regulation, water-electrolyte balance, and hormone secretion are primarily mediated through AGTR1 (Vervoort et al., 2002). Adrenomedullin (ADM), a peptide hormone with widespread expression across various tissues, is implicated in a diverse range of physiological processes, including the regulation of hormonal secretion, glucose metabolism, and the inflammatory response (Wong et al., 2014). In liver sinusoidal endothelial cells (LSECs), homeostatic adaptation to systemic iron overload involves the transcriptional induction of Bone Morphogenetic Protein 6 (BMP6) (Charlebois et al., 2023). Depletion of IRS2 impairs cell proliferation and reduces hormone secretion in mouse granulosa cells (Lei et al., 2018). EPHRIN-A5 is essential for maintaining optimal fertility and eliciting a full ovulatory response to gonadotropins in female mice (Buensuceso et al., 2016). The Rabphilin-3A-like (RPH3AL) protein plays a pivotal role in regulating hormone exocytosis (Chen et al., 2011). The elevated expression of these genes suggests that the regulation of coagulation, hemostasis, hormone transport, hormone secretion, and blood coagulation are principal biological processes occurring in the liver of 6-month-old human embryo. Notably, this increased gene expression indicates that the liver may effectively regulate blood coagulation and maintain hemostatic balance. It appears that the liver possesses the capacity to regulate blood coagulation and hemostasis through hormone secretion at this stage of development.

In comparison to the liver of 6-month-old human embryo, the upregulated genes associated with the biological process of steroid metabolic process, such as *CYP26A1*, *APOL1*, *SULT2A1*, *PLEKHA1*, *UGT2B10*, *APOA4*, *BMP5*, *PDE8B*, *LEPR*, *NR5A2*, *PROX1*, *FGF23*, *EGR1*, *INSIG2*, *BMP2*, *APOL2*, *CYP2E1*, *UGT2A3* were identified in the liver of 7-month-old human embryo (Figure 5E). CYP26A1 plays a crucial role in hepatic function by regulating retinoic acid metabolism (Wang et al., 2021). SULT2A1 preferentially acts on hydroxysteroids, including dehydroepiandrosterone, testosterone/dihydrotestosterone, and pregnenolone, as well as on amphipathic sterol bile acids derived from cholesterol (Chatterjee et al., 2005). APOA4 is involved in lipid transport and metabolism by modulating hormonal regulation (Nazzari et al., 2024). PDE8B regulates basal corticosterone synthesis through both acute and chronic mechanisms (Tsai and Beavo, 2011). Polymorphisms in the LEPR gene are associated with type 2 diabetes and related metabolic traits in a Chinese population (Zhang et al., 2018). The increased expression of these genes indicate that the liver of 7-month-old human embryo likely possesses the capability to regulate metabolic processes associated with steroids. This observation suggests a potential functional maturity in the enzymatic systems of the embryonic liver involved in steroid metabolism. Further research into this phenomenon could provide essential insights into the emergence of liver functions during human development and the molecular mechanisms governing metabolic pathways of fetal maturation. Additionally, the biological processes of complement activation, blood coagulation, cellular carbohydrate metabolism, serine family amino acid metabolism, myeloid leukocyte activation, humoral immune response, T cell proliferation, T cell differentiation, response to peptide hormone, cellular response to peptide hormone stimulus, steroid metabolic process and response to steroid hormone were identified along the pseudotime from 3-month-old to 7-month-old (Figure 6A). These findings align with previously reported data, demonstrating that distinct biological processes occur in the liver at various developmental stages. Collectively, these results suggest that the embryonic liver possesses not only hematopoietic functions but also other biological functions that emerge and evolve throughout the embryonic period.

Finally, the genes associated with lymphocyte differentiation, such as *TNFSF13B*, *CD79A*, *ITK*, *LEPR*, *EGR1*, *PTPN22*, *IL6*, *CD8A*, *FZD7*, *MS4A1*, *CD1D*, *EOMES*, *NFIL3*, *GPR183*, *ONECUT1*, *PTGER4* exhibited high expression levels in the liver of 7-month-old human embryo (Figure 7B). B cell-activating factor (BAFF), also known as CD257, TNFSF13B, and BLyS, is recognized as a critical regulator of B cell development and differentiation (Tangye et al., 2006). CD79a is expressed on both normal and neoplastic B cells and is considered B-cell-specific (Pilozzi et al., 1998). Interleukin-2 Tyrosine Kinase (ITK) plays a crucial role in the differentiation of T helper 9 (Th9) cells through Interleukin-2 (IL-2) and Interferon Regulatory Factor 4 (IRF4) (Gomez-Rodriguez et al., 2016). EGR1 and NFAT2 have been shown to cooperatively regulate the expression of the expression of the regulatory factor ID3 and the recombinase RAG2, both of which are essential for T-lymphocyte differentiation (Koltsova et al., 2007). PTPN22 encodes lymphoid-specific tyrosine phosphatase LYP, a non-receptor type protein tyrosine phosphatase. LYP is critical for lymphocyte activation and differentiation (Gianchecchi et al., 2013). NFIL3 is essential for the development of all innate lymphoid cell subsets (Seillet et al., 2014). Additionally, the genes associated with the regulation of hemopoiesis, including *CYP26B1*, *TBX21*, *LAG3*, *SFRP1*, *IL4I1*, *PTN*, *VNN1*, *LOX*, *CD80*, *TNFAIP6*, *INHA*, *PCK1*, *IRF1*, *IL2RA*, *IL10*, *IRF4*, *CTLA4*, *IL15*, *TLR3*, *TNFRSF11B*, *MALT1*, *TRIB1*, *GPR171*, *TCIM*, *EGR3*, *CIB1*, *IRF7*, *ZFP36L1*, *ISG15*, *HLA-DRB1* were identified in the liver of 7-month-old human embryo (Figure 7B). SFRP1 plays a vital role in maintaining hematopoietic stem cell (HSC) homeostasis by externally regulating β-catenin signaling (Renstrom et al., 2009). Loss of IRF1 impairs HSC self-renewal, increases stress-induced cell cycle entry, and confers resistance to apoptosis (Rundberg Nilsson et al., 2023). Tribbles pseudokinases, consisting of TRIB1, TRIB2, and TRIB3, are crucial regulators of both normal and malignant hematopoiesis (Salome et al., 2018). GPR171, identified as a putative P2Y-like receptor, is involved in the negative regulation of myeloid differentiation in murine hematopoietic progenitor cells (Rossi et al., 2013). TCIM (C8orf4) exerts regulatory control over hematopoietic stem and progenitor cells, as well as hematopoiesis (Jung et al., 2014). ZFP36L1 enhances monocyte/macrophage differentiation by repressing CDK6 (Chen et al., 2015). IRF7 inhibits hematopoietic regeneration under stress by targeting CXCR4 (Chen et al., 2021). The genes associated with liver development, such as *HGF*, *CPS1*, *OTC*, *PROX1*, *ASS1*, *GATA6* were significantly upregulated in the liver of 7-month-old human embryo (Figure 7B). PROX1 is essential for hepatocyte migration during liver development. A deficiency in *PROX1* results in a reduced-size liver, characterized by a diminished population of clustered hepatocytes (Sosa-Pineda et al., 2000). Hepatocyte growth factor (HGF) is clinically significant in liver fibrosis, hepatocyte regeneration post-inflammation, and post-transplant liver regeneration (Zhao et al., 2022). GATA6 is involved in various stages of liver development, including endoderm liver-lineage determination, liver specification, hepatic bud outgrowth, and hepatoblast differentiation (Zhang and He, 2018). Building on these findings, Figure 7A illustrates the gene interaction network associated with regulation of hemopoiesis, lymphocyte differentiation and liver development. Analysis of this network reveals complex interactions among genes, underscoring the necessity of their coordinated expression for typical liver maturation. The delineation of this network has provided significant insights into the coordinated molecular activities essential for the appropriate development and functionality of the embryonic liver. Understanding these gene interactions is imperative for elucidating the complexities of embryonic liver development and its diverse physiological roles.

## Conclusion

In this study, the gene expression profiles in the liver across various development stages of human embryos were determined. Genes with high expression levels were enriched in the biological processes such as humoral immune response, B cell mediated immunity, response to vitamin and brown fat cell differentiation in the livers of 3-month-old and 4-month-old human embryos. As development progressed, the genes involved in T cell differentiation, coagulation, hormone transport, T cell activation and steroid metabolic process were upregulated in the liver. These results indicate that immune response and hematopoiesis are significant processes in the early embryonic liver. As the embryonic liver matures, it progressively acquires metabolic functions. These results suggest that the liver in embryogenesis not only possesses hematopoietic function but also develops other biological functions that emerge and evolve throughout the embryonic period. Moreover, this study identified not only genes previously associated with liver development but also a plethora of uncharacterized genes that exhibit high expression levels relevant to liver development. These insights will establish a groundwork for investigating fetal liver development and pathologies linked to embryonic liver abnormalities.

## Data Availability

The datasets presented in this study can be found in online repositories. The names of the repository/repositories and accession number(s) can be found in the article/supplementary material.

## Supplementary material

SUPPLEMENTARY FIGURE S1 The genes along the pseudotime from the 3-month-old to 7-month-old liver. **(A)** The expression of PROZ and FGG along the pseudotime from the 3-month-old to 4-month-old liver. **(B)** The expression of DDIT4 1020 and ACACB along the pseudotime from the 4-month-old to 5-month-old liver. **(C)** The expression of SYK, PIK3R1, SPI1, PTPRC, SOX4, DOCK2, ANXA1 along the pseudotime from the 5-month-old to 6-month-old liver. **(D)** The expression of UGT2B7, TIPARP, PROX1, PLEKHA1, LEPR, INSIG2, CYP19A1, NR5A2 along the pseudotime of the 7-month-old liver.

SUPPLEMENTARY FIGURE S2 The genes with low expression levels and biological processes in the liver of various developmental stages. **(A)** GO analysis of the down-regulated genes identified in the liver of 3-month-old human embryo. **(B)** KEGG pathway analysis of the down-regulated genes identified in the liver of 3-month-old human embryo. **(C)** GO analysis of the down-regulated genes identified in the liver of 4-month-old human embryo. **(D)** KEGG pathway analysis of the down-regulated genes identified in the 4-month-old human embryo. **(E)** GO analysis of the down-regulated genes identified in the liver of 5-month-old human embryo. **(F)** KEGG pathway analysis of the down-regulated genes identified in the liver of 5-month-old human embryo. **(G)** GO analysis of the down-regulated genes identified in the liver of 6-month-old human embryo. **(H)** KEGG pathway analysis of the down-regulated genes identified in the liver of 6-month-old human embryo. **(I)** GO analysis of the down-regulated genes identified in the liver of 7-month-old human embryo. **(J)** KEGG pathway analysis of the down-regulated genes identified in the liver of 7-month-old human embryo.

SUPPLEMENTARY FIGURE S3 The expression analysis of genes associated with the biological processes of the regulation of hemopoiesis, liver development and lymphocyte differentiation in the liver of various developmental stages. **(A)** Heatmaps illustrate the expression profiles of genes related to the regulation of hemopoiesis, liver development and lymphocyte differentiation between the livers of 2-month-old and 3-month-old human embryos. **(B)** Heatmaps illustrate the expression profiles of genes related to the regulation of hemopoiesis, liver development and lymphocyte differentiation between the livers of 3-month-old and 4-month-old human embryos. **(C)** Heatmaps illustrate the expression profiles of genes related to the regulation of hemopoiesis, liver development and lymphocyte differentiation between the livers of 4-month-old and 5-month-old human embryos. **(D)** Heatmaps illustrate the expression profiles of genes related to the regulation of hemopoiesis, liver development and lymphocyte differentiation between the livers of 5-month-old and 6-month-old human embryos.
