# Supplementary figures and images for: Study on gene expression in the liver at various developmental stages of human embryos
Source: Front Cell Dev Biol. 2025 Jan 8;12:1515524. doi: 10.3389/fcell.2024.1515524 (PMC11751009; doi:10.3389/fcell.2024.1515524)

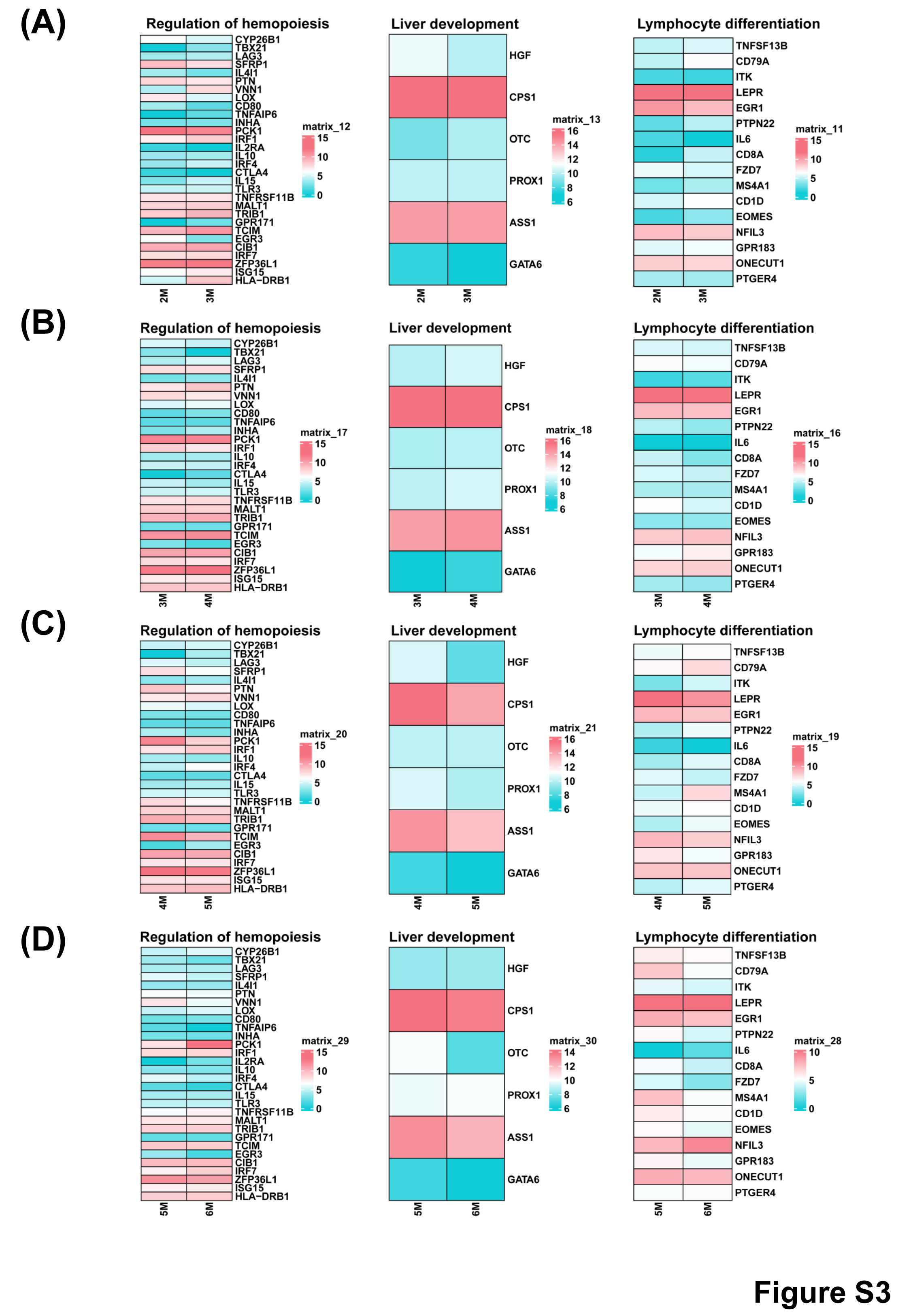

Supplement: Supplementary file 1 [file Image3.tif]

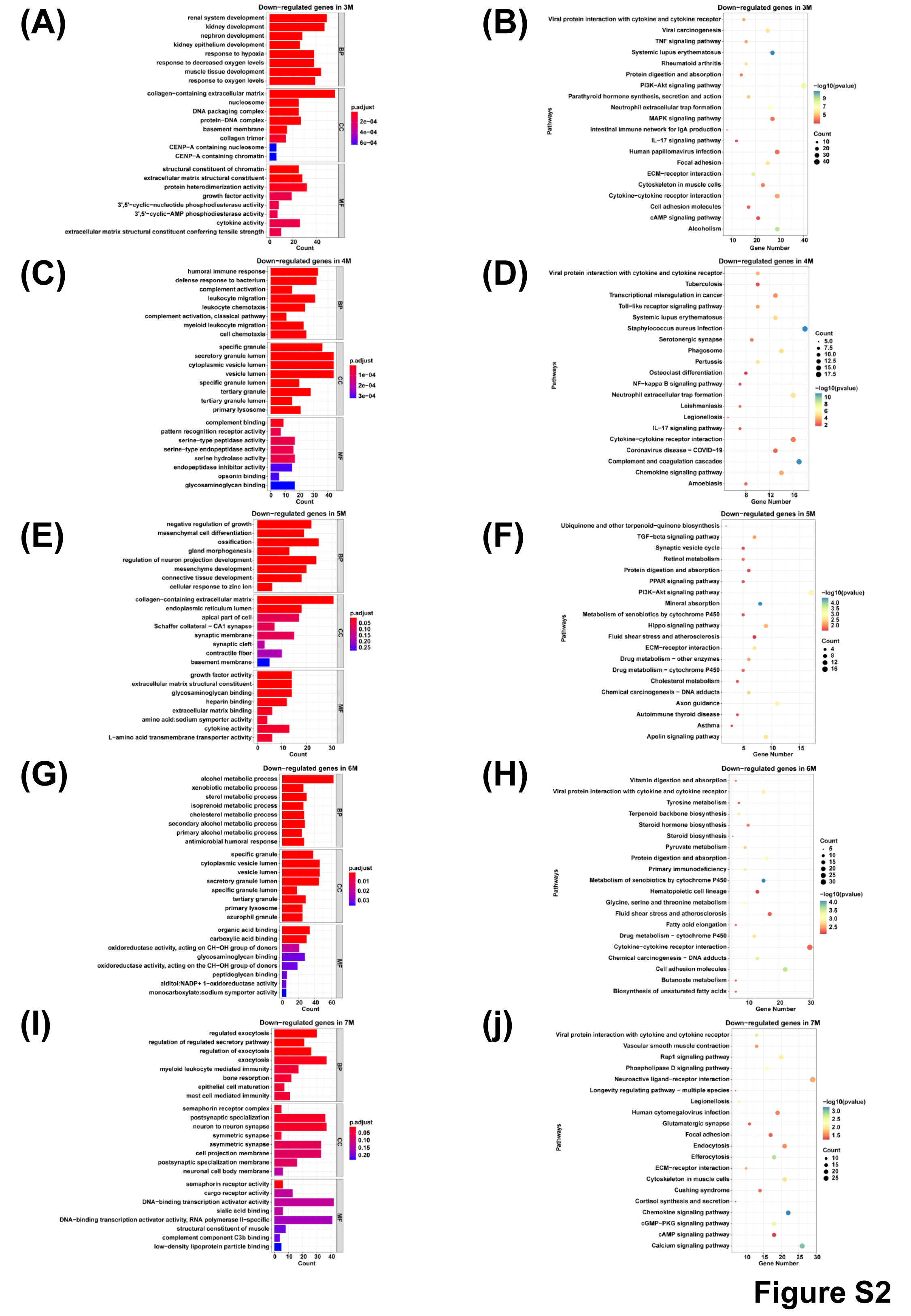

Supplement: Supplementary file 2 [file Image2.tif]

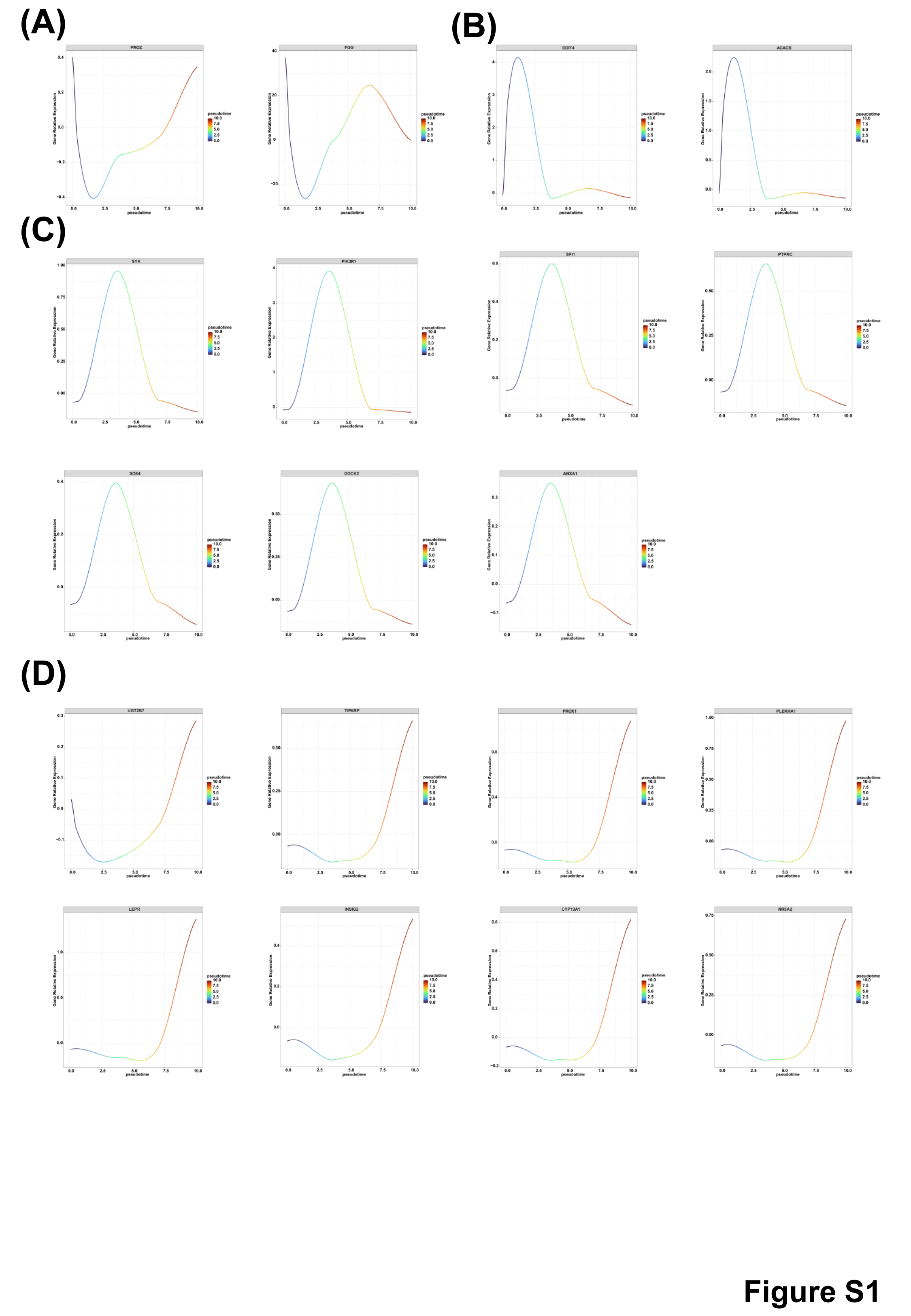

Supplement: Supplementary file 3 [file Image1.tif]
